# Detection and targeting insulin growth factor receptor type 2 (IGF2R) in osteosarcoma PDX in mouse models and in canine osteosarcoma tumors

**DOI:** 10.1038/s41598-019-47808-y

**Published:** 2019-08-07

**Authors:** Sharayu Karkare, Kevin J. H. Allen, Rubin Jiao, Mackenzie E. Malo, Wojciech Dawicki, Muath Helal, Dale L. Godson, Ryan Dickinson, Valerie MacDonald-Dickinson, Rui Yang, Bang Hoang, Richard Gorlick, David S. Geller, Ekaterina Dadachova

**Affiliations:** 1 University of Saskatchewan, Saskatoon, Canada; 2 Western College of Veterinary Medicine, Saskatoon, Canada; 3 Montefiore Medical Center and the Children’s Hospital at Montefiore, The University Hospital for Albert Einstein College of Medicine, Bronx, NY USA; 4 MD Anderson Cancer Center, Houston, TX USA

**Keywords:** Molecular medicine, Targeted therapies

## Abstract

Osteosarcoma (OS) represents 3.4% of all childhood cancers with overall survival of 70% not improving in 30 years. The consistent surface overexpression of insulin-like growth factor-2 receptor (IGF2R) has been reported in commercial and patient-derived xenograft (PDX) OS cell lines. We aimed to assess efficacy and safety of treating PDX and commercial OS tumors in mice with radiolabeled antibody to IGF2R and to investigate IGF2R expression on canine OS tumors. IGF2R expression on human commercial lines 143B and SaOS2 and PDX lines OS-17, OS-33 and OS-31 was evaluated by FACS. The biodistribution and microSPECT/CT imaging with 111Indium-2G11 mAb was performed in 143B and OS-17 tumor-bearing SCID mice and followed by radioimmunotherapy (RIT) with 177Lutetium-2G11 and safety evaluation. IGF2R expression in randomly selected canine OS tumors was measured by immunohistochemistry. All OS cell lines expressed IGF2R. Biodistribution and microSPECT/CT revealed selective uptake of 2G11 mAb in 143B and OS-17 xenografts. RIT significantly slowed down the growth of OS-17 and 143B tumors without local and systemic toxicity. Canine OS tumors expressed IGF2R. This study demonstrates the feasibility of targeting IGF2R on OS in PDX and spontaneous canine tumors and sets the stage for further development of RIT of OS using comparative oncology.

## Introduction

Despite its relative rarity, osteosarcoma (OS) remains the most common primary malignant bone tumor, with about 900 new cases reported within the United States per year (American Cancer Society) representing about 3.4% of all childhood cancers^1^. It is the fifth most common primary malignancy among adolescents and young adults^2^ and the most common non-hematologic bone cancer^3^. Unfortunately, overall survival remains stagnant at approximately 70%, and treatment has not evolved in a meaningful way in over 30 years^4,5^. Regardless of therapy, patients who exhibit overt metastatic disease continue to suffer dismal outcomes, with metastases to the lungs and to the bone portending an overall survival of less than 40% and 20%, respectively. Unlike some cancers that share a common genetic driver, OS demonstrates tremendous genetic variability^6^, precluding the use of more conventional targeted therapies and illustrating the need for alternate approaches.

The cation independent mannose-6-phosphate/insulin-like growth factor-2 receptor (IGF2R) normal function is the sequestration of IGF-2 to prevent pro-mitogenic IGF signalling, and transport of lysosomal acid hydrolase precursors from the Golgi to the lysosome. Recently, the consistent surface overexpression of the cation independent mannose-6-phosphate/insulin-like growth factor-2 receptor (IGF2R) has been reported across a panel of osteosarcoma tumors, including both standard and patient-derived xenograft lines^7^. Moreover, a single nucleotide polymorphism (SNP) within a haplotype block in IGF2R was previously linked to an increased osteosarcoma risk^8^. Further investigation of IGF2R in the context of OS is warranted, while present findings suggest that IGF2R appears to be involved in, and perhaps essential, to the development of OS, making it a promising therapeutic target.

Targeted radionuclide therapy (TRT) delivers cytocidal radiation in the form of alpha- or beta-particle emitting radionuclides to the tumor with high precision, thus avoiding many of the side effects associated with external beam radiation therapy (EBRT). Recent regulatory approvals of ^223^Radium chloride (Xofigo) for treatment of prostate cancer metastasized to the bone, and of ^177^Lutetium-labeled peptide (Lutathera) for treatment of somatostatin receptor-positive gastroenteropancreatic neuroendocrine tumors (GEP-NETs), attests to the great promise and flexibility of TRT. Radioimmunotherapy (RIT) is a subset of TRT and is a method of delivering cytotoxic radiation in a targeted fashion whereby an antigen-specific antibody is bound to either an alpha- or beta-emitting radioisotope^9,10^. RIT was regulatory approved more than a decade ago for refractory and recurrent non-Hodgkin’s lymphoma (Zevalin)^11^ as well as has been successfully employed in a number of clinical trials^10^. RIT delivers cytocidal radiation to the targeted cells independently of any specific pathway and is unaffected by resistance mechanisms or tumor microenvironmental fluctuations. It permits for systemic administration, antibody-mediated specificity, and physical cytocidal damage in a manner well tolerated by the patient.

Previously, we have used a xenograft OS mouse model to demonstrate the preferential localization of an IGF2R-radiolabeled monoclonal antibody (mAb) to tumor when compared with its isotype control. Additionally, we have shown that treatment using ^188^Rhenium (^188^Re)-labeled IGF2R-specific mAb MEM-238 resulted in growth inhibition and possibly growth regression^12^. This was accomplished using a single non-optimized dose and compared against multiple controls, serving as a proof of concept. Limitations of this study included the inherent properties of MEM-238, which was designed to bind only human IGF2R and thus limited the assessment of mouse tissue binding. Additionally, only a single patient-derived xenograft tumor line was used. Recently, a novel murine mAb (2G11) that binds both human and murine IGF2R became commercially available. The goals of the current study were: (1) to evaluate the biodistribution of the 2G11 mAb using multiple tumor lines within an OS xenograft model; (2) perform pilot RIT of OS with 2G11 mAb radiolabeled with beta-emitter ^177^Lutetium (^177^Lu); (3) to assess 2G11 ability to bind to IGF2R in canine OS tumors from companion dogs as a prelude to demonstrating the efficacy and safety of RIT in animal models of compelling translational significance.

## Results

### OS cell lines expressed various levels of IGF2R accompanied by internalization of the mAb to IGF2R

**Figure 1:**
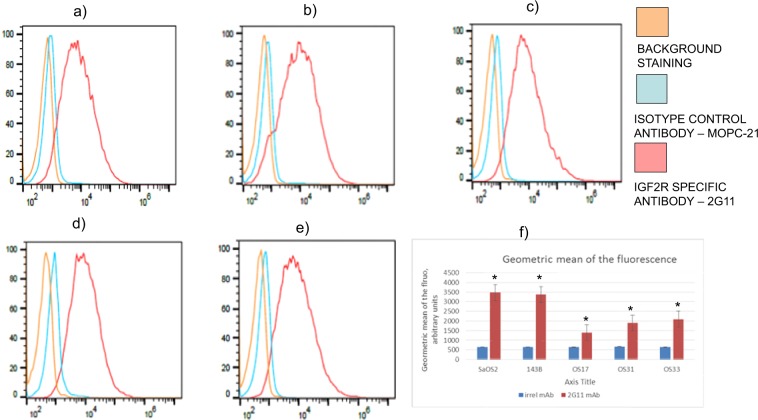
Flow cytometry results of mAb 2G11 binding to live OS cells: (**a**) 143B; (**b**) SaOS2; (**c**) OS-33; (**d**) OS-31; (**e**) OS-17; (**f**) comparative plot. Control mAb MOPC-21 and cells without the antibody were used as negative controls. *Denotes significant difference when compared to MOPC-21 binding.

**Figure 2:**
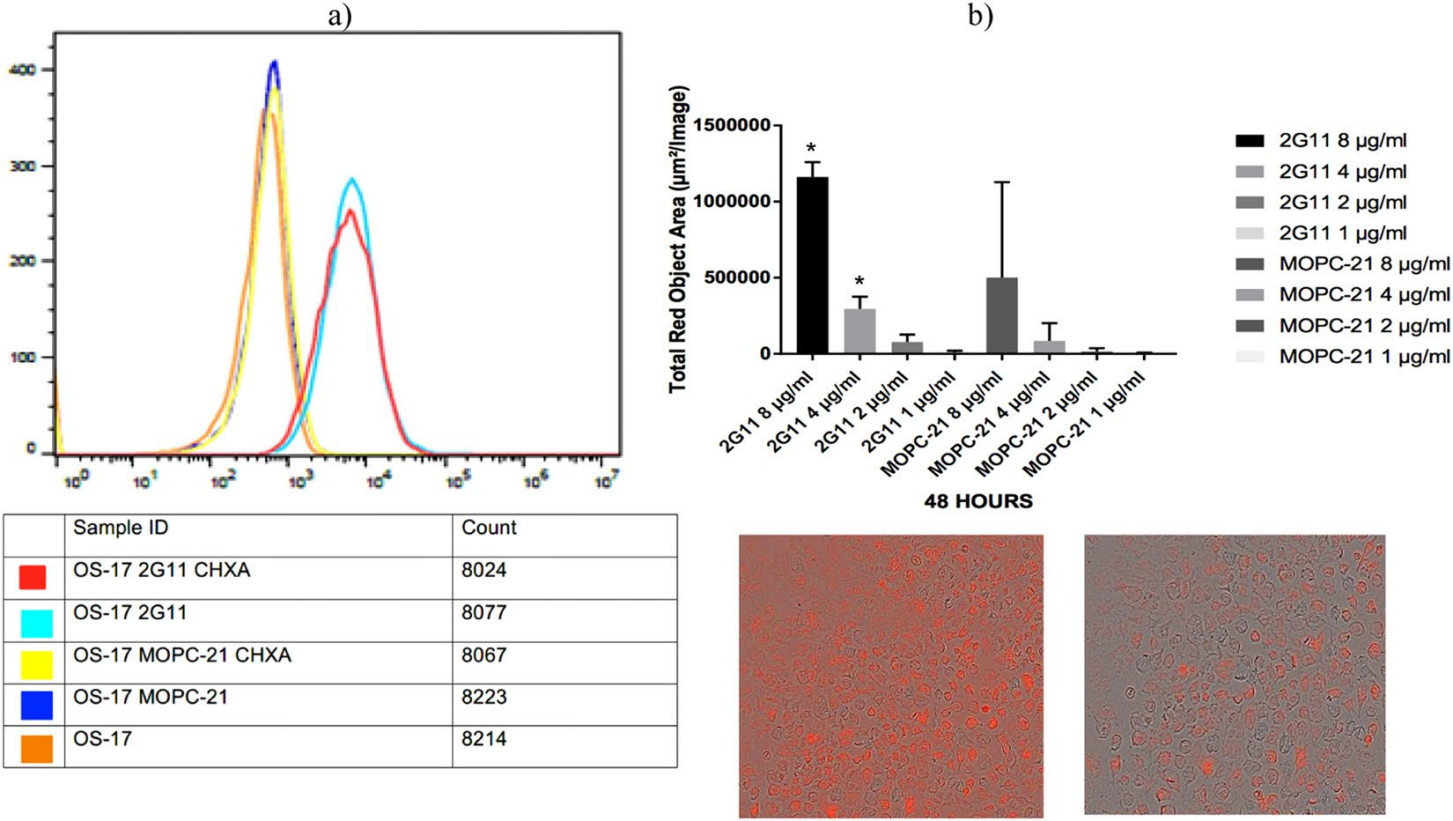
Evaluation of binding of CHXA”-conjugated 2G11 mAb to OS-17 IGF2R-expressing cells by flow cytometry and internalization of 2G11 mAb into 143B cells: (**a**) flow cytometry results. Naked 2G11 was used as a positive control; control mAb MOPC-21, CHXA” conjugated MOPC21, and OS17 cells were used as negative controls; (**b**) internalization results. 2G11 and MOPC-21 mAbs were conjugated to IncuCyte FabFluor Red Antibody Labeling Reagent and internalization into 143B OS cells was analyzed at 48 hrs post-binding. Insert on the left shows 143B cells incubated with 2G11 mAb and on the right – with irrelevant MOPC-21 mAb. There was significantly more internalization of 2G11 at 48 hrs (P = 0.02, denoted with*).

2G11 mAb specifically binds to IGF2R-expressing cell lines and preserves its immunoreactivity post-conjugation of the bifunctional chelating agent. We first sought to establish if 2G11 mAb to human and murine IGF2R would specifically bind to OS across multiple cell lines. Flow cytometry was utilized to analyze the expression of IGF2R across two standard cell lines (143B, SaOS2) and three patient-derived xenograft tumors (OS-17, OS-31, OS-33). The 2G11 mAb specifically bound to all 5 tumor lines when compared with its isotype control, MOPC-21 (Fig. 1a–e). The expression of IGF2R by standard cells lines SaOS2 and 143B was 1.5–2 times higher than that of the patient-derived xenograft tumors. Of the patient-derived cell lines, OS-17 demonstrated lower IGF2R expression then did OS-33 (Fig. 1f). The highest IGF2R-expressing line, 143B, and the lowest IGF2R-expressing line, OS-17, were selected for use in subsequent biodistribution and RIT experiments. To enable the radiolabeling of 2G11 mAb with ^111^In and ^177^Lu for the biodistibution and therapeutic studies, 2G11 was conjugated to the bifunctional chelating agent CHXA”. Flow cytometry analysis showed that CHXA”-conjugated 2G11 successfully bound OS-17 cells to the same extent as naked 2G11 mAb (Fig. 2a).

Antibody internalization contributes to the retention of the radionuclides in the tumor. For this reason we evaluated the internalization of 2G11 mAb in 143B cells using pH sensitive dye which is not fluorescent at neutral pH but become highly fluorescent at acidic pH. When an antibody labeled with the pH sensor fluorescent dye binds to its antigen on cancer cells, it does not become fluorescent but when internalization into the endosomal and lysosome vehicles takes place the pH drops and the dye becomes fluorescent. It follows the process from early endosomes (pH 6.3) to lysosomes (pH 4.7). Antibody internalization experiment demonstrated significant internalization of 2G11 mAb within 48 hrs of being added to OS (P = 0.02), in contrast to the non-specific control MOPC-21 mAb which showed minimal internalization (Fig. 2b).

### Biodistribution and microSPECT/CT imaging revealed selective uptake of 2G11 mAb in OS xenografts and significant IGF2R expression in the spleens of SCID mice

**Figure 3:**
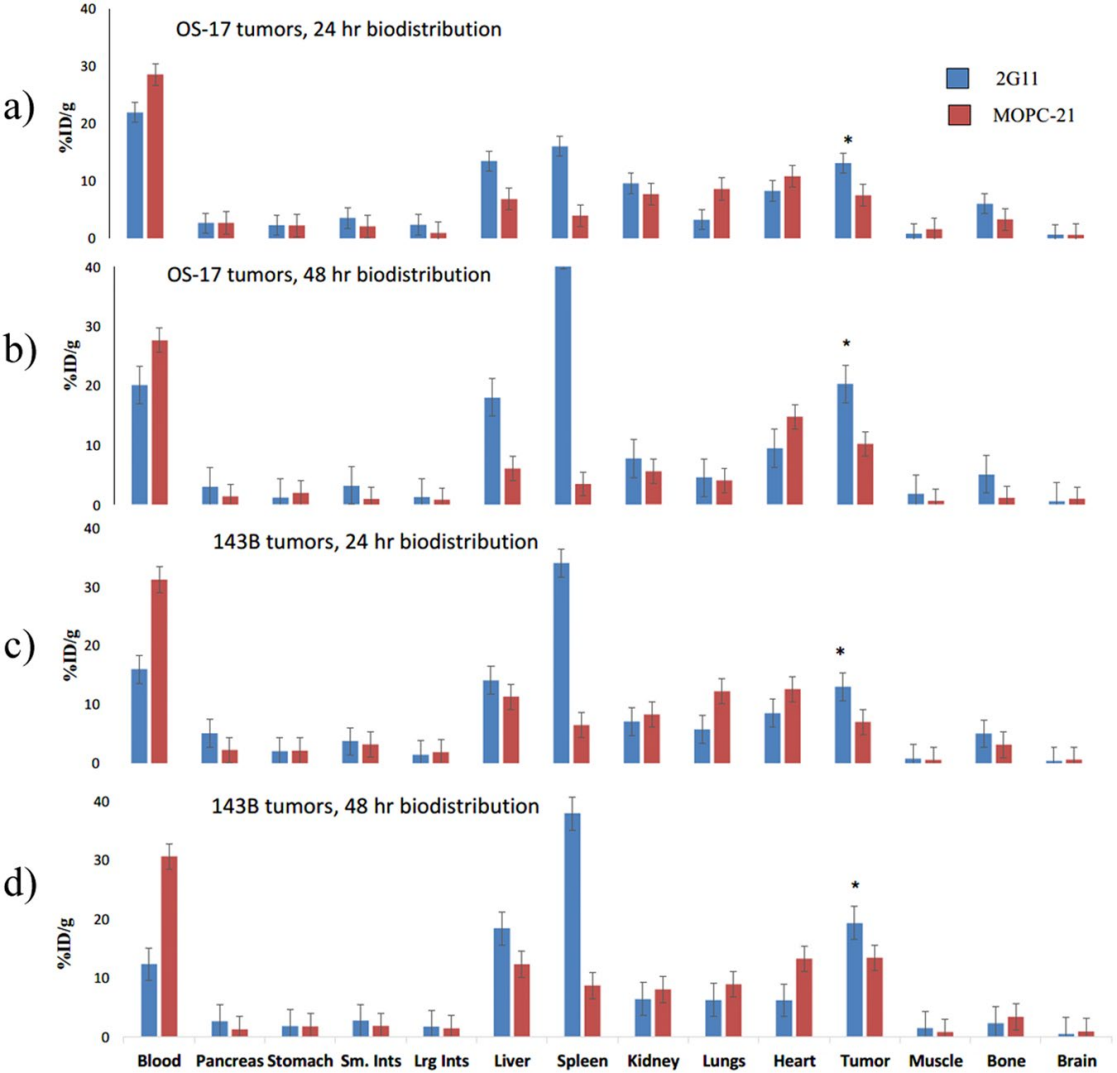
Biodistribution of 2G11 mAb labeled with ^111^In in 143B and OS-17 tumor-bearing SCID mice: (**a**,**b**) OS-17 at 24 and 48 hrs post antibody administration, respectively; (**c**,**d**) 143B at 24 and 48 hrs post antibody administration, respectively. The uptake in the tumors at 24 and 48 hours was significantly higher for 2G11 mAb than for MOPC-21 for both cell lines (P = 0.03, denoted with*).

**Figure 4:**
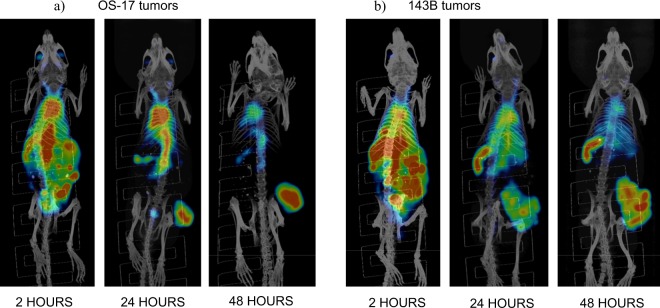
microSPECT/CT imaging of SCID mice bearing OS tumors with ^111^In-2G11 mAb: (**a**) OS-17 tumors; (**b**) 143B tumors.

**Figure 5:**
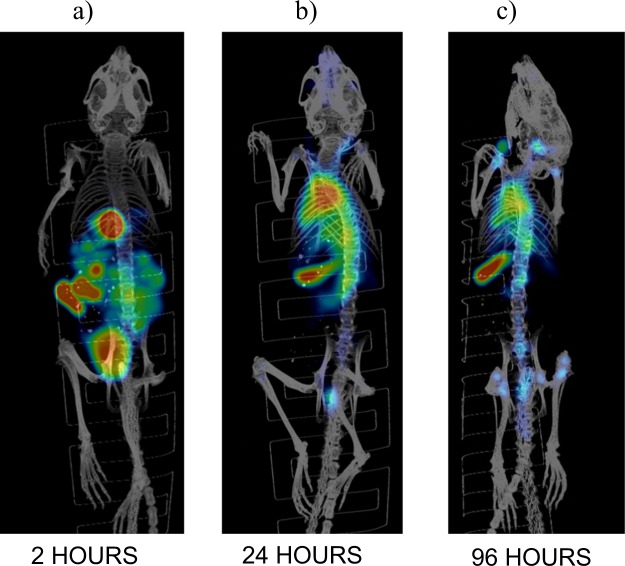
microSPECT/CT imaging of healthy SCID mice with ^111^In-2G11 mAb: (**a**) 2 hrs, (**b**) 24 hrs, (**c**) 96 hrs post administration of ^111^In-2G11.

To determine if 2G11 mAb is capable of selectively targeting IGF2R-expressing OS tumors, this mAb and the isotype matching murine mAb MOPC-21 were radiolabeled with ^111^In and administered IP to 143B and OS-17 tumor-bearing mice. The mice were sacrificed at 24 and 48 hrs after the administration of the radiolabeled mAbs and a biodistribution was performed (Fig. 3). The uptake in the tumors at 24 and 48 hours was significantly higher for 2G11 mAb than for MOPC-21 for both cell lines (P = 0.03). Interestingly, *in vitro* 143B cells expressed approximately 2 times more IGF2R than OS-17 cells, whereas *in vivo* the uptake of IGF2R-binding 2G11 mAb into the tumors was similar for both cells lines, with the tumor uptake being even slightly higher, though not significantly, in OS-17 tumors. There was several-fold higher uptake of 2G11 in the spleens of mice with both tumor types than that of MOPC-21, which was unremarkable. Following the biodistribution experiments, we performed microSPECT/CT imaging of tumor-bearing mice with ^111^In-2G11 mAb to confirm the biodistribution results in living animals. Figure 4a shows the microSPECT imaging of OS-17 tumor-bearing mice, and Fig. 4b - of 143B tumor-bearing mice at 2, 24 and 48 hrs post administration of the radiolabeled 2G11 mAb. While at 2 hrs post administration the majority of the mAb was still in circulation with tumors practically not visible, at 24 hrs the tumors were clearly visualized, and the spleens became visible as well. By 48 hrs the mAb cleared almost completely from the circulation with only the tumors and the spleens retaining the high amounts of the 2G11 mAbs. To investigate if the high spleen uptake of 2G11 was due to the possible metastatic spread of tumor cells into the spleen, we performed microSPECT/CT imaging of the healthy SCID mice with ^111^In- 2G11 mAb at 2, 24, and 96 hrs post mAb administration (Fig. 5). High spleen uptake was seen on the images starting at 24 hrs, confirming that the 2G11 mAb is binding to the IGF2R expressed by the spleen cells of SCID mice.

### RIT with radiolabeled 2G11 mAb significantly slowed down the growth of OS-17 and 143B OS tumors

**Figure 6:**
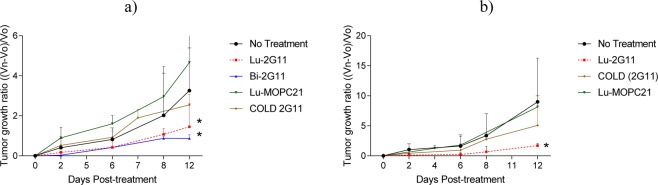
Radioimmunotherapy of OS tumor-bearing SCID mice: (**a**) 143B tumors; (**b**) OS-17 tumors. The mice were given IP either 80 μCi ^177^Lu-2G11, or 80 μCi ^177^Lu-MOPC-21, or unlabeled (cold) 2G11, or was left untreated. A group of 143B tumor bearing mice were also given 80 μCi ^213^Bi-2G11. Administration of ^177^Lu-2G11 significantly (P < 0.05, denoted with *) slowed down the growth of both 143B (**a**) and OS-17 tumors (**b**) in comparison with the untreated tumors and “cold” 2G11. The treatment effect was IGF2R-specific as the control mAb MOPC-21 radiolabeled with the same activity of ^177^Lu, had significantly less effect on the tumor growth rate (P = 0.06).

To investigate if radiolabeling 2G11 with therapeutic radionuclides will confer therapeutic properties, we radiolabeled it with an intermediate energy beta-emitter ^177^Lu. Pilot experiments demonstrated that the maximum tolerated dose for ^177^Lu-2G11 is below 250 μCi, and in the follow up experiments the mice were given IP either 80 μCi ^177^Lu-2G11, 80 μCi ^177^Lu-MOPC-21, unlabeled (cold) 2G11, or were left untreated. Administration of ^177^Lu-2G11 significantly (P < 0.05) slowed down the growth of both 143B (Fig. 6a) and OS-17 tumors (Fig. 6b) in comparison with the untreated tumors and “cold” 2G11. The treatment effect was IGF2R-specific as the control mAb MOPC-21 radiolabeled with the same activity of ^177^Lu, had significantly less effect on the tumor growth rate (P = 0.06). In addition, a group of mice with 143B tumors received 80 μCi of the alpha-emitter ^213^Bi-2G11 mAb, and its retardation effect on the tumor growth became more profound, though not significantly, than that of ^177^Lu-2G11 by day 12 post treatment (P = 0.055). The tumors in all groups started to develop ulcerations by day 12 and necessitated euthanizing the animals in compliance with the Animal Research Ethics regulation.

### RIT with radiolabeled 2G11 did not cause local or systemic toxicity

**Figure 7:**
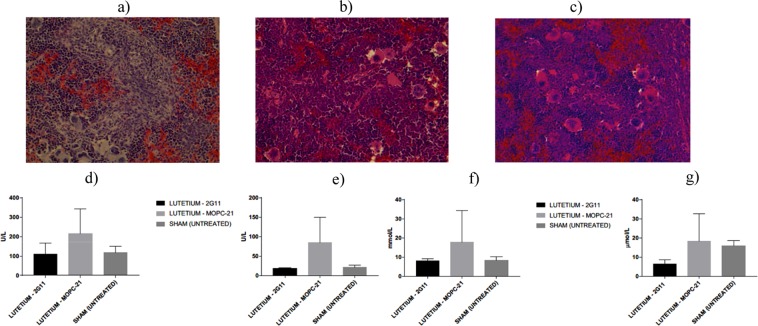
Evaluation of the side effects of RIT of OS tumor-bearing SCID mice. (**a–c**) H&E staining of spleens from the mice treated with: (**a**) untreated; (**b**) ^177^Lu-2G11; (**c**) ^213^Bi-2G11; (**d**–**g**) evaluation of the systemic toxicity: (**d**) AST; (**e**) ALT; (**f**) BUN; (**g**) creatinine.

Histological splenic analysis from untreated and treated mice was performed at the completion of the experiments to assess whether high uptake of the radiolabeled 2G11 in the spleen resulted in detectable injury. Findings demonstrate that neither ^177^Lu-2G11 nor ^213^Bi-2G11 produced histologically overt splenic damage (Fig. 7a–c). The levels of liver enzymes (ALT and AST) as well as of creatinine and BUN were within the normal range for SCID mice (Fig. 7d–g) attesting to the high tolerability of RIT.

### OS tumors from companion dogs expressed IGF2R

**Figure 8:**
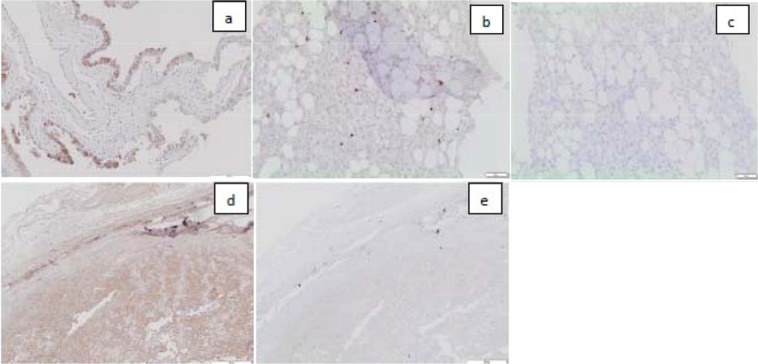
Immunohistochemistry of the canine OS tumors with 2G11 mAb: (**a**) canine placenta as a positive control. IGF2R-positive epithelial cells are stained brown; (**b**) OS from a companion dog (case D14-26248) stained with 2G11 mAb; (**c**) the same tumor stained with the control MOPC-21 mAb; (**d**) OS from a companion dog (case D15-06705) stained with 2G11 mAb; (**e**) the same tumor stained with the control MOPC21 mAb.

We investigated the 2G11 mAb binding to canine OS using immunohistochemistry from two randomly selected companion dogs from a cohort treated at Western College of Veterinary Medicine (WCVM) at the University of Saskatchewan (Fig. 8). The murine mAb MOPC-21 was used as a non-specific control and canine placenta was used as positive control for 2G11 (Fig. 8a). OS tumor cells robustly stained using the IGF2R-specific 2G11 mAb for both cases (Fig. 8b,d). No staining was noted using the control mAb MOPC-21.

## Discussion

This study demonstrates the feasibility of targeting OS using a commercially available mAb to IGF2R within both a xenograft murine and a spontaneous canine model. Biodistribution and microSPECT/CT imaging reveal selective uptake of the radiolabeled IGF2R-specific mAb 2G11 within the tumor as well as the spleen, with the latter resulting in no detectable untoward effect within the experimental time course. Moreover, the treatment using both the beta emitting ^177^Lu-2G11 mAb conjugate and the alpha emitting ^213^Bi-2G11 mAb conjugate showed substantially slowed tumor growth *in vivo* using both a standard cell line and a PDX tumor line. No local or systemic toxicity was noted. Finally, tumors from two randomly sampled companion canines revealed IGF2R expression, suggesting that further comparative studies may offer valuable clinical insight and merit consideration.

Use of the beta-emitting radionuclide, ^177^Lu (maximum beta energy 0.13 MeV) offers multiple benefits over ^188^Re, which was previously utilized. Firstly, ^177^Lu has a longer physical half-life of 6.7 days, extending the therapeutic effect for a longer period. Secondly, the process of labeling ^177^Lu is simpler and more reliable^13^. Finally, ^177^Lu is readily available from several vendors, likely is due to the recent approval of the ^177^Lu-labeled peptide (Lutathera) in Europe and the United States for the treatment of somatostatin receptor positive neuroendocrine tumors. Importantly, our laboratory has recently demonstrated the equivalency of ^188^Re- and ^177^Lu-labeled mAbs in the treatment of experimental cervical cancer^14^. Taken together, the use of ^177^Lu appears to support the treatments’ technical feasibility and clinical translation. Additionally, while previous work has demonstrated tight Ab binding to IGF2R, Scatchard plot analysis has shown only a moderate number of available IGF2R binding sites. Antibodies labeled with alpha emitters are, at least theoretically, less dependent on the antigen density as one or two hits of an alpha-particle can kill a cell^9^.

The biodistribution and microSPECT/CT imaging experiments in the two OS xenograft models showed high IGF2R-specific uptake of ^111^In-2G11 in the tumors that was not dependent on the *in vitro* levels of IGF2R expression by the respective cell line, nevertheless, it was IGF2R-specific, as control mAb MOPC-21 bound much less to the tumors in both models. This observation suggests that even tumors with lower IGF2R expression will be effectively targeted with the IGF2R-specific mAb. The uptake of ^111^In-2G11 into the tumors at both 24 and 48 hrs was significantly higher than the previously observed uptake of ^188^Re-MEM-238^12^. This might be due to: (1) internalization of 2G11 mAb upon binding to IGF2R as demonstrated by internalization experiments (Fig. 2b); (2) the residualizing nature of trivalent radiometals such as ^111^In or ^177^Lu that tend to remain in the tumor even after separating from the mAb, as opposed to ^188^Re, which leaves the tumors relatively fast after oxidizing into the inert perrhenate anion. The high uptake of radiolabeled 2G11 mAb into tumors translated into an impressive therapeutic response for both OS-17 and 143B xenograft models. Interestingly, a very short-lived alpha-emitter ^213^Bi (46 min physical half-life versus 6.7 days for ^177^Lu) had a similar effect to ^177^Lu treated mice for the 143B tumor xenografts, highlighting the therapeutic potential of alpha-emitters in RIT.

While systemic chemotherapy combined with wide surgical resection remains the mainstay of OS treatment^5^, there remains no meaningful alternative therapy for those patients who relapse following standard first-line therapy. The addition of conventional cytotoxic chemotherapy to the standard three-drug backbone consisting of high-dose methotrexate, doxorubicin and cisplatin, has failed to increase overall survival. This stark reality underscores the need for innovative disruptive approaches to OS therapy, particularly for patients who present with overt metastases or who relapse following standard therapy. While OS has been historically considered a radiation-resistant histology, the entire radiation oncology field has enjoyed numerous advances and improvements. Currently, the application of radiation therapy has been well-established in specific instances. For example, patients unable to undergo an R0 resection, particularly in the spine and in the pelvis, benefit from the addition of external beam radiation, realizing improved outcomes when compared with chemotherapy alone^15,16^. External beam radiation also offers a well-tolerated palliative option in cases where cure is no longer feasible and where the main treatment objective shifts to improving pain control and quality of life.

Radionuclide therapy has been utilized for the management of metastatic disease to the bone in both the setting of carcinoma and OS. Both Samarium-153 and Radium-223 localize to areas of increased bone turnover, thereby delivering relatively high doses of selective treatment and yielding a reduction in pain and a decrease in analgesia dependence in a number of prospective double-blinded placebo-controlled randomized studies^17–20^. However, these radiopharmaceuticals are only used for the palliation of painful bone metastases, and are not viewed as standard of care. Combination of radionuclide and monoclonal antibodies is a recognized strategy used for a number of hematopoietic and solid tumors, combining the targeted specificity of the monoclonal antibody with cytotoxic impact exerted by the radionuclide^10^. This approach may offer further benefit via the abscopal effect, a well-recognized phenomenon, described as a radiation-mediated tumor response in disease outside of or distant from the field of radiation.

Limitation of the current study includes the relatively short experimental time course, which resulted from the need to euthanize the animals owing to local tumor ulceration despite tumor response. Both the natural course of a growing tumor and treatment-related event could contribute to ulceration and further characterization of tumor histology may offer insight. The use of immunocompromised animals is an inherent limitation in that it discounts or artificially nulls any immune-mediated response. For this reason and despite being a recognized model, it simply does not recapitulate the human condition. Planned utilization of murine OS within an immunocompetent murine model was not feasible, given the lack of IGF2R overexpression within murine OS. Taken together, these limitations illustrate the need for improved preclinical models which allow for the evaluation of human tumor within immune-intact animals.

The observation of high spleen uptake of ^111^In-2G11 during biodistribution and microSPECT/CT imaging of OS tumor-bearing and healthy SCID mice raised the concern about potential undesirable effects of RIT on this organ and may represent a translational limitation despite the histological splenic analysis which failed to demonstrate any evidence of organ injury. As with all therapeutic measures, safety remains a paramount and critical consideration. In addition to the normal splenic histology, no abnormalities in the liver enzymes and kidney biomakers such as creatinine and BUN were detected. It has been shown by Laube^21^ that 2G11 mAb was able to mimic the effect of mannose-6-phosphate on the receptor up-regulation. As 2G11 stays in circulation for several days (Figs 5 and 6), it upregulates the IGF2R expression in the tumor and the spleen, which results in its increased uptake in those tissues. Interestingly, according to^21^ mAb MEM-238 which was also used in our initial RIT of OS experiments^12^ does not upregulates IGF2R. This observation can explain why the tumor uptake of radiolabeled MEM-238 at 48 hrs was only 3.5% ID/g, while the uptake of 2G11 at 48 hrs in the current study was 5 times higher at 18% ID/g. In regard to the clinical translation,  according to the Human Atlas Database, IGF2R is only minimally or moderately expressed in all normal organs in humans, suggesting that using it as a target for RIT should be relatively safe: https://www.proteinatlas.org/ENSG00000197081-IGF2R/tissue. For comparison, SSTR1, which is targeted by the recently approved Lutathera, is highly expressed in several crucial organs and is still safe to administer to patients: https://www.proteinatlas.org/ENSG00000139874-SSTR1/tissue. The safety aspects of RIT targeting IGF2R should be carefully evaluated in the future clinical trials.

Safety can be further demonstrated within the context of companion animals, which serve as a great resource, as shown by the remarkable growth of comparative oncology over the last 30 years^22^. In this regard, canine and human OS share certain antigens and can be targeted with the same mAbs^23^, and the mAbs to human cation independent mannose-6-phosphate receptor also bind to canine one^24^. In fact, a sequence alignment of human, mouse and canine IGF2R genes encompassing the IGFII binding region (containing domains 11-FNII), shows that this region is highly conserved across these species with 82% sequence identity. Randomly selected cases of canine OS from the companion dogs treated in our institution, demonstrated significant expression of IGF2R. As there are overwhelming similarities between human and canine osteosarcomas^25^ - presence of IGF2R in canine tumors sets the stage of further development of IGF2R-targeting RIT of OS using the comparative oncology approach.

## Materials and Methods

### Reagents and antibodies

2G11 and MOPC21 mAbs were obtained from ThermoFisher (Canada). (R)-2-Amino-3-(4-isothiocyanatophenyl)propyl]-trans-(S,S)-cyclohexane-1,2-diamine-pentaacetic acid (CHXA”) BCA was purchased from Macrocyclics (USA). ^111^In was obtained from Nordion (Canada); ^177^Lu was purchased from RadioMedix (USA); and ^225^Ac/^213^Bi generator – from Oak Ridge National Laboratory (USA). Silica gel instant thin layer chromatography (SG-iTLC) strips were obtained from Agilent (Canada).

### Cell lines

Human osteosarcoma cell lines 143B and SaOS2 were obtained from American Type Culture Collection (ATCC, Manassas, VA, USA). OS-17, OS-33 and OS-31, well characterized patient-derived osteosarcoma cells lines are maintained in our laboratories. Cell lines were cultured in Eagle’s Minimum Essential medium and supplemented with 10% FBS, sodium pyruvate, non-essential amino acids, and 100 U penicillin/0.1 mg/ml streptomycin.

### Flow cytometry

The binding efficiency of the IGF2R-specific mAb 2G11 to live OS cells was assessed by flow cytometry with the purpose of selecting cell lines with the lowest and highest IGF2R expression for biodistribution. Both IGF2R specific 2G11 and isotype matching control MOPC-21 mAbs were used at the concentration of 1 μg/μl.

### Antibody internalization assay

143B OS cells in Minimum Essential medium were seeded at a concentration of 4000–5000 cells/well in 50 μL volume into a 96-well flat bottom microplate and were left to adhere and grow for 24 hours. The IncuCyte FabFluor Red Antibody Labeling Reagent was rehydrated with 100 μL sterile water to result in a final concentration of 0.5 mg/ml. 2G11 and MOPC-21 in Eagle’s Minimum Essential medium (EMEM) were mixed in microcentrifuge tubes protected from light with the IncuCyte FabFluor Red Antibody Labeling Reagent at a molar ratio of 1:3. The mixture was incubated for 15 min at 37 °C in the dark to allow for conjugation, then added to the cells at the mAbs concentration range of 1–8 μg/ml, and the plate was placed into the IncuCyte Live-cell Analysis System. Images were captured by every 15 min with 10X objective for 48 hrs. During the analysis the Red fluorescent channel was turned on and the background fluorescence was turned off by using the background subtraction feature.

### Animal models

All animal studies were approved by the Animal Research Ethics Board of the University of Saskatchewan (#2017006). All animal experiments were performed in accordance with the Canadian Council on Animal Care guidelines for humane animal use. Six-eight week old SCID (CB17/Icr-*Prkdc*^*scid*^/IcrIcoCrl) female mice obtained from Charles River Laboratories (USA) were anesthetized with isoflurane and injected subcutaneously with 3 × 10^6^ of either OS-17 or 143B cells into the right flank. Mice were monitored for tumor development, and it was noted that for the 143B cell line 80% of mice developed palpable tumors by day 12, and for the OS-17 cell line - by day 5.

#### Conjugation of bifunctional chelating agent CHXA” to mAB’s

10X conjugation buffer (0.05 M Carbonate/Bicarbonate, 0.15 M NaCl, 5 mM EDTA, pH 8.6–8.7), 5 mL was combined with 0.5 M EDTA, pH = 8.0 (0.5 mL) and was diluted to 50 mL in a 50 mL Falcon tube with deionized water to give the 1X buffer. An Amicon Ultra 0.5 mL centrifugal filter (30 K MW cut off, Fisher) was loaded with 2 mg of either 2G11 or MOPC-21 antibody. The antibody was exchanged into the above conjugation buffer by performing 6 × 1.5 mL washes using an Amicon concentrator in a refrigerated centrifuge at 4 °C. A solution of bifunctional CHXA” ligand with 2 mg/mL concentration was prepared by dissolving CHXA” in conjugation buffer. The antibody was recovered from the Amicon and 23.6 µL of 2 mg/mL CHXA” solution in conjugation buffer is added to provide 5 fold molar excess of CHXA” over the antibody. The reaction mixture was incubated at 37 °C for 1.5 hrs. The reaction mixtures was then purified into 0.15 M ammonium acetate buffer, pH = 6.5–7.0, with 6 × 1.5 mL washes on Amicon concentrators in a refrigerated centrifuge at 4 °C. The sample were stored at 4 °C. A Bradford assay was performed to determine protein recovery and concentration.

### Radiolabeling of antibodies

The IGF2R-specific mAb 2G11 and the isotype matching control mAb MOPC-21 were labeled with the radioisotopes ^111^In (^111^In-2G11, ^111^In-MOPC-21), ^177^Lu (^177^Lu-2G11, ^177^Lu-MOPC-21) or ^213^Bi (^213^Bi-2G11). The radiolabeling of an antibody-CHXA” conjugate with ^111^In was performed to achieve the specific activity of approximately 5 µCi/µg of the antibody. For example, 600 µCi of ^111^In chloride was added to 10 µL 0.15 M ammonium acetate buffer and added to a microcentrifuge tube containing 120 µg 2G11-CHXA” conjugate in 0.15 M ammonium acetate buffer. The reaction mixture was incubated for 60 min at 37 °C, and then the reaction was quenched by the addition of 3 µL 0.05 M EDTA solution. The percentage of radiolabeling was measured by SG-iTLC using 0.15 M ammonium acetate buffer as the eluent. SG-iTLCs were cut in half and read on a Perkin Elmer 2470 Automatic Gamma Counter (top containing unlabeled ^111^In, bottom containing antibody conjugated ^111^In). ^177^Lu labeling was performed with an identical protocol using ^177^Lu as the radioisotope, and labeling with ^213^Bi was carried out as in^26^. ^111^In, ^177^Lu and ^213^Bi incorporation was >95%.

### Biodistribution study

The biodistribution was performed to assess whether IGF2R-specific mAb 2G11 will localize in the tumor preferentially when compared to non-specific isotopes matching control MOPC-21. Tumors were allowed to grow until they reached an average size of 70–100 mm^3^. The volume was calculated assuming elliptical geometry and using the following formula: V = (LxW^2^)/2. Tumor bearing mice were randomized into the groups of 5 animals and intraperitoneal (IP) injected with 20 μCi of either ^111^In-2G11, or ^111^In-MOPC-21. At 24 and 48 hr post injection 5 mice were sacrificed from each group: 143B tumor bearing mice injected with ^111^In- 2G11 or MOPC-21, and OS-17 tumor bearing mice injected with ^111^In- 2G11 or MOPC-21. Once sacrificed the tumor, blood, heart, lungs, pancreas, spleen, kidney, liver, brain, a section of the small and large intestines, thigh muscle, and femur were collected, weighed, and counted in a gamma counter (Perkin Elmer). The percent of injected dose per gram (%ID/g) for each sample was calculated.

#### MicroSPECT/CT Imaging

microSPECT/CT (micro single photon emission computer tomography/computer tomography) images were collected on a MILabs VECTor^4^ (Netherlands) microSPECT/CT scanner and processed using the comprehensive image analysis software package PMOD (version 3.9, PMOD Technologies, Inc, Switzerland). Imaging studies were conducted using 200 μCi ^111^In at a 5:1 mCi/mg specific activity with a CHXA” conjugated 2G11. Tumor-bearing mice were administered ^111^In-2G11 via IP injection and imaged in the prone position at 2, 24, and 48 hours post injection. SPECT data was collected for 20 minutes using an Extra Ultra High Sensitivity Mouse (XUHS-M) collimator for 20–350 keV range using spiral trajectories. All SPECT images were reconstructed using both 245 keV and 171 keV ^111^In gamma emissions on a 0.4 mm voxel grid with MILabs reconstruction software.

### RIT study

OS-17 and 143B tumor bearing mice were monitored until the tumor size reached 70–100 mm^3^. The mice were randomized into groups of 5 mice per group. Group 1 received 80 μCi ^177^Lu-2G11, group 2–80 μCi ^177^Lu-MOPC-21, group 3 - unlabeled (cold) 2G11, and group 4 was left untreated. In addition, a group of mice with 143B tumors was given 80 μCi ^213^Bi-2G11. Tumors were monitored as previously described and average tumor volume was calculated for the mice in each group over a 12-day period. At the completion of the observation period the mice were humanely sacrificed, their spleens removed and analyzed histologically for signs of radiation damage. Their blood was analyzed for AST, ALT, BUN and creatinine as biomarkers of possible liver and kidney toxicity.

### Histology of the mouse spleens and canine osteosarcoma tumors

The spleens from the mice were harvested at the conclusion of the RIT study, fixed in 4% paraformaldehyde, decalcified, and embedded in paraffin blocks. Paraffin-embedded tissue slides were heated at 60 °C for 1 hr, deparaffinized using xylenes and rehydrated using graded alcohols. The spleens were stained with hematoxylin and eosin (H&E). Immunohistochemical detection of IGF2R in canine OS tumors was performed using an automated staining platform (Autostainer Plus, Dako Canada Inc., Mississauga, ON). Endogenous peroxidase activity was quenched using 3% hydrogen peroxide in methanol. Heat-induced epitope retrieval was performed in a Tris/EDTA pH 9 buffer for 20 min. The tissue was incubated with 1:25 dilution of 2G11 mAb overnight at 4 °C. Bound primary antibody was detected using an HRP-labelled polymer detection reagent (EnVision + System, Dako Canada Inc., Mississauga, ON) with 3,3′-diaminobenzidine tetrahydrochloride (DAB) (Dako Canada Inc., Mississauga, ON) as the chromogen and a hematoxylin counterstain. Canine paraffin-embedded placenta was used as the positive control, and MOPC-21 IgG1 was used instead of the primary antibody as the isotype negative control.

### Statistical analyses

The differences between the biodistribution groups were analyzed using the Kruskal–Wallis and/or the Mann–Whitney tests. Differences between the treatment groups were analyzed using two way ANOVA test.

## Data Availability

The authors will make materials, data and associated protocols promptly available to readers upon publication in Scientific Reports without undue qualifications in materials transfer agreements. The data that support the findings of this study are available from the corresponding author upon reasonable request.

## References

[1] Gurney, J. G., Swensen, A. R. & Bulterys, M. Malignant bone tumors. In: *Cancer Incidence and Survival Among Children and Adolescents: United States SEER Program 1975*–*1995*, Pub *#99-4649* (eds Ries, L. A., Smith, M. A. S. & Gurney, J. G.) 99–110 (National Cancer Institute, Bethesda, MD, 1999).

[2] Smith, M. A., Gurney, J. G. & Ries, L. A. Cancer in adolescents 15 to 19 years old. In: *Cancer Incidence and Survival Among Children and Adolescents: United States SEER Program 1975–1995*, Pub *#99-4649* (eds Ries, L. A., Smith, M. A. S. & Gurney, J. G.) 110–115 (National Cancer Institute, Bethesda, MD, 1999).

[3] Mirabello L, Troisi RJ, Savage SA (2009). International osteosarcoma incidence patterns in children and adolescents, middle ages and elderly persons. Int J Cancer..

[4] Meyers PA (2005). Osteosarcoma: A randomized, prospective trial of the addition of ifosfamide and/or muramyl tripeptide to cisplatin, doxorubicin, and high-dose methotrexate. J Clin Oncol.

[5] Ferrari S (2005). Neoadjuvant chemotherapy with high-dose Ifosfamide, high-dose methotrexate, cisplatin, and doxorubicin for patients with localized osteosarcoma of the extremity: A joint study by the Italian and Scandinavian Sarcoma Groups. J Clin Oncol.

[6] Ladanyi M, Gorlick R (2000). Molecular pathology and molecular pharmacology of osteosarcoma. Pediatr Pathol Lab Med.

[7] Hassan SE (2012). Cell surface receptor expression patterns in osteosarcoma. Cancer.

[8] Savage SA (2007). National Osteosarcoma Etiology Study Group. Analysis of genes critical for growth regulation identifies Insulin-like Growth Factor 2 Receptor variations with possible functional significance as risk factors for osteosarcoma. Cancer Epidemiol Biomarkers Prev..

[9] Milenic DE, Brady ED, Brechbiel MW (2004). Antibody-targeted radiation cancer therapy. Nature Rev Drug Discovery.

[10] Larson SM, Carrasquillo JA, Cheung NV, Press OW (2015). Radioimmunotherapy of human tumours. Nature.

[11] Kaminski MS (2005). 131I-tositumomab therapy as initial treatment for follicular lymphoma. N Engl J Med.

[12] Geller DS (2016). Targeted therapy of osteosarcoma with radiolabeled monoclonal antibody to an insulin-like growth factor-2 receptor (IGF2R). Nucl. Med. Biol..

[13] Severi S (2017). Peptide receptor radionuclide therapy in the management of gastrointestinal neuroendocrine tumors: efficacy profile, safety, and quality of life. Onco Targets Ther..

[14] Phaeton R (2016). Beta emitters Rhenium-188 and Lutetium-177 are equally effective in radioimmunotherapy of HPV-positive experimental cervical cancer. Cancer Med..

[15] Ozaki T (2002). Osteosarcoma of the spine: experience of the Cooperative Osteosarcoma Study Group. Cancer.

[16] Ozaki T (2003). Osteosarcoma of the pelvis: experience of the Cooperative Osteosarcoma Study Group. J Clin Oncol..

[17] Serafini AN (1998). Palliation of pain associated with metastatic bone cancer using samarium-153 lexidronam: a double-blind placebo-controlled clinical trial. J Clin Oncol..

[18] Sartor O (2004). Quadramet 424Sm10/11 Study Group. Samarium-153-Lexidronam complex for treatment of painful bone metastases in hormone-refractory prostate cancer. Urology.

[19] Sartor O (2014). Effect of radium-223 dichloride on symptomatic skeletal events in patients with castration-resistant prostate cancer and bone metastases: results from a phase 3, double-blind, randomised trial. Lancet Oncol..

[20] Parker C (2013). Alpha emitter radium-223 and survival in metastatic prostate cancer. N Engl J Med..

[21] Laube F (2009). Mannose-6-phosphate/insulin-like growth factor-II receptor in human melanoma cells: effect of ligands and antibodies on the receptor expression. Anticancer Res..

[22] Khanna C (2016). The current state and a perspective towards the future of osteosarcoma in dogs. Vet Comp Oncol..

[23] Haines DM, Bruland OS (1989). Immunohistochemical detection of osteosarcoma-associated antigen in canine osteosarcoma. Anticancer Res..

[24] Prydz K, Brändli AW, Bomsel M, Simons K (1990). Surface distribution of the mannose 6-phosphate receptors in epithelial Madin-Darby canine kidney cells. J Biol Chem..

[25] Withrow SJ, Wilkins RM (2010). Cross talk from pets to people: translational osteosarcoma treatments. ILAR J..

[26] McFarren A (2016). A fully human antibody to gp41 selectively eliminates HIV-infected cells that transmigrated across a model human blood brain barrier. AIDS.

